# Supplementary material for: Analysis of International Coexistence Management of Genetically Modified and Non-Genetically Modified Crops
Source: Plants (Basel). 2025 Mar 13;14(6):895. doi: 10.3390/plants14060895 (PMC11944432; doi:10.3390/plants14060895)
Supplement: Supplementary file 1 [file plants-14-00895-s001.zip › plants-3302275-supplementary.pdf]

Supplementary Table S1. GM varieties approved in China.

	Name of variety	Name of transformant	Transferred genes	GM traits
GM maize	yufeng303D	DBN9936	*cry1Ab, epsps*	Resistant to Ostrinia furnacalis and glyphosate
GM maize	zhongkeyu505D	DBN9936	*cry1Ab, epsps*	Resistant to Ostrinia furnacalis, armyworms and glyphosate herbicides
GM maize	jiexi100D	DBN9936	*cry1Ab, epsps*	Resistant to Ostrinia furnacalis and glyphosate
GM maize	zhongkeyu505R	ruifeng125	*cry1Ab/cry2Aj, g10evo-epsps*	Resistant to Ostrinia furnacalis
GM maize	yufeng303R	ruifeng125	*cry1Ab/cry2Aj, g10evo-epsps*	Resistant to Ostrinia furnacalis
GM maize	yufeng303H	DBN9858	*epsps, pat*	Resistant to glyphosate and glufosinate
GM maize	jingke968TK	ruifeng125	*cry1Ab/cry2Aj, g10evo-epsps*	Resistant to Ostrinia furnacalis
GM maize	jingke968D	DBN9936	*cry1Ab, epsps*	Resistant to Ostrinia furnacalis and glyphosate
GM maize	zhengdan958D	DBN9936	*cry1Ab, epsps*	Resistant to Ostrinia furnacalis and glyphosate
GM maize	nonhghua803D	DBN9936	*cry1Ab, epsps*	Resistant to Ostrinia furnacalis and glyphosate
GM maize	nongda372R	ruifeng125	*cry1Ab/cry2Aj, g10evo-epsps*	Resistant to Ostrinia furnacalis, armyworms, cotton bollworm
GM maize	zhengdan958K	ND207	*mcry1Ab, mcry2Ab*	Resistant to Ostrinia furnacalis
GM maize	ruipu909D	DBN9936	*cry1Ab, epsps*	Resistant to Ostrinia furnacalis, armyworms and glyphosate herbicides
GM maize	dafeng30F	DBN9936	*cry1Ab, epsps*	Resistant to Ostrinia furnacalis, armyworms and glyphosate herbicides
GM maize	lihe1D	DBN9936	*cry1Ab, epsps*	Resistant to Ostrinia furnacalis, armyworms and glyphosate herbicides
GM maize	kehe699D	DBN9936	*cry1Ab, epsps*	Resistant to Ostrinia furnacalis, armyworms and glyphosate herbicides
GM maize	dongdan1331D	DBN9936	*cry1Ab, epsps*	Resistant to Ostrinia furnacalis, armyworms and glyphosate herbicides
GM maize	dongdan1331K	ND207	*mcry1Ab, mcry2Ab*	Resistant to Ostrinia furnacalis
GM maize	hongshuo899SK	DBN9936	*cry1Ab, epsps*	Resistant to Ostrinia furnacalis and glyphosate
GM maize	xiangyu998HZ	ruifeng125	*cry1Ab/cry2Aj, g10evo-epsps*	Resistant to Ostrinia furnacalis

	Name of variety	Name of transformant	Transferred genes	GM traits
GM maize	youdi919HZ	ruifeng125	*cry1Ab/cry2Aj, g10evo-epsps*	Resistant to Ostrinia furnacalis
GM maize	tianyu108Z	ND207	*mcry1Ab, mcry2Ab*	Resistant to Ostrinia furnacalis
GM maize	zengyu1572KK	DBN9936	*cry1Ab, epsps*	Resistant to Ostrinia furnacalis and glyphosate
GM maize	denghai605D	DBN9936	*cry1Ab, epsps*	Resistant to Ostrinia furnacalis and glyphosate
GM maize	denghai533D	DBN9936	*cry1Ab, epsps*	Resistant to Ostrinia furnacalis and glyphosate
GM maize	zhengdan958GK	ruifeng125	*cry1Ab/cry2Aj, g10evo-epsps*	Resistant to Ostrinia furnacalis, armyworms
GM maize	jinyuanyu177K	ND207	*mcry1Ab, mcry2Ab*	Resistant to Ostrinia furnacalis
GM maize	jingke986GE	ruifeng125	*cry1Ab/cry2Aj, g10evo-epsps*	Resistant to Ostrinia furnacalis, armyworms
GM maize	kangnong20065 KK	DBN9936	*cry1Ab, epsps*	Resistant to Ostrinia furnacalis, armyworms, cotton bollworm and glyphosate
GM maize	huimin207R	ruifeng125	*cry1Ab/cry2Aj, g10evo-epsps*	Resistant to Ostrinia furnacalis, armyworms
GM maize	yuanke105WG	Bt11×GA21	*cry1Ab, pat, mepsps*	Resistant to Ostrinia furnacalis, armyworms and glyphosate herbicides
GM maize	yuanke105D	DBN9936	*cry1Ab, epsps*	Resistant to Ostrinia furnacalis, armyworms and glyphosate herbicides
GM maize	heyu187D	DBN9936	*cry1Ab, epsps*	Resistant to Ostrinia furnacalis, armyworms and glyphosate herbicides
GM maize	xianda901ZL	Bt11×MIR162×GA21	*cry1Ab, pat, vip3Aa20*	Resistant to Ostrinia furnacalis, armyworms, cotton bollworm, Spodoptera frugiperda, glyphosate and glufosinate glyphosate
GM maize	tie391K	DBN9936	*cry1Ab, epsps*	Resistant to Ostrinia furnacalis, armyworms and glyphosate herbicides
GM maize	luodan566DT	DBN3601T	*cry1Ab, epsps, vip3Aa19, pat*	Resistant to Ostrinia furnacalis and glyphosate
GM maize	wugu3861KK	DBN3601T	*cry1Ab, epsps, vip3Aa19, pat*	Resistant to Ostrinia furnacalis, armyworms, cotton bollworm, glyphosate and glufosinate glyphosate
GM maize	hengfeng728D	DBN9936	*cry1Ab, epsps*	Resistant to Ostrinia furnacalis and glyphosate
GM maize	leying797D	DBN9936	*cry1Ab, epsps*	Resistant to Ostrinia furnacalis, armyworms and glyphosate herbicides

	Name of variety	Name of transformant	Transferred genes	GM traits
GM maize	shengmei999D	DBN9936	*cry1Ab, epsps*	Resistant to Ostrinia furnacalis, armyworms and glyphosate herbicides
GM maize	yufeng623K	ND207	*mcry1Ab, mcry2Ab*	Resistant to Ostrinia furnacalis, armyworms
GM maize	tiannongjiuD	DBN9936	*cry1Ab, epsps*	Resistant to Ostrinia furnacalis, armyworms and glyphosate herbicides
GM maize	tianyu616K	ND207	*mcry1Ab, mcry2Ab*	Resistant to Ostrinia furnacalis, armyworms
GM maize	liaoke38K	ND207	*mcry1Ab, mcry2Ab*	Resistant to Ostrinia furnacalis, armyworms
GM maize	liaoke38D	DBN9936	*cry1Ab, epsps*	Resistant to Ostrinia furnacalis and glyphosate
GM maize	jingke968K	ND207	*mcry1Ab, mcry2Ab*	Resistant to Ostrinia furnacalis, armyworms
GM maize	jinkeyu3306D	DBN9936	*cry1Ab, epsps*	Resistant to Ostrinia furnacalis and glyphosate
GM maize	huxin358D	DBN9936	*cry1Ab, epsps*	Resistant to Ostrinia furnacalis, armyworms and glyphosate herbicides
GM maize	rongyu8K	ND207	*mcry1Ab, mcry2Ab*	Resistant to Ostrinia furnacalis, armyworms
GM maize	hongkai706D	DBN9936	*cry1Ab, epsps*	Resistant to Ostrinia furnacalis, armyworms, cotton bollworm and glyphosate
GM maize	hengyu1D	DBN9936	*cry1Ab, epsps*	Resistant to Ostrinia furnacalis, armyworms and glyphosate herbicides
GM maize	longken1755K	ND207	*mcry1Ab, mcry2Ab*	Resistant to Ostrinia furnacalis, armyworms
GM maize	aomei95D	DBN9936	*cry1Ab, epsps*	Resistant to Ostrinia furnacalis, armyworms and glyphosate herbicides
GM maize	heyu185WG	Bt11×GA21	*cry1Ab, pat, mepsps*	Resistant to Ostrinia furnacalis, armyworms
GM maize	lianchuang808R	ruifeng125	*cry1Ab/cry2Aj, g10evo-epsps*	Resistant to Ostrinia furnacalis
GM maize	jingnongke728K	ND207	*mcry1Ab, mcry2Ab*	Resistant to Ostrinia furnacalis, armyworms, cotton bollworm
GM maize	dedan123R	ruifeng125	*cry1Ab/cry2Aj, g10evo-epsps*	Resistant to Ostrinia furnacalis, armyworms, cotton bollworm
GM maize	nongda778D	DBN9936	*cry1Ab, epsps*	Resistant to Ostrinia furnacalis, armyworms, cotton bollworm and glyphosate
GM maize	liangyu99D	DBN9936	*cry1Ab, epsps*	Resistant to Ostrinia furnacalis, armyworms, cotton bollworm and glyphosate

	Name of variety	Name of transformant	Transferred genes	GM traits
GM maize	longping218R	ruifeng125	*cry1Ab/cry2Aj, g10evo-epsps*	Resistant to Ostrinia furnacalis
GM maize	denghai533K	ND207	*mcry1Ab, mcry2Ab*	Resistant to Ostrinia furnacalis, armyworms
GM maize	denghai685D	DBN9936	*cry1Ab, epsps*	Resistant to Ostrinia furnacalis, armyworms, cotton bollworm and glyphosate
GM maize	denghai710D	DBN9936	*cry1Ab, epsps*	Resistant to Ostrinia furnacalis, armyworms, cotton bollworm and glyphosate
GM maize	huaxingdan88DT	DBN3601T	*cry1Ab, epsps, vip3Aa19, pat*	Resistant to Ostrinia furnacalis, armyworms, cotton bollworm, glyphosate and glufosinate glyphosate
GM soybean	maiyu526	DBN9004	*epsps, pat*	Resistant to glyphosate and glufosinate
GM soybean	maiyu503	DBN9004	*epsps, pat*	Resistant to glyphosate and glufosinate
GM soybean	maiyu511	DBN9004	*epsps, pat*	Resistant to glyphosate and glufosinate
GM soybean	maiyu579	DBN9004	*epsps, pat*	Resistant to glyphosate and glufosinate
GM soybean	maiyu565	DBN9004	*epsps, pat*	Resistant to glyphosate and glufosinate
GM soybean	zhongliandou1505	zhonghuang6106	*g2-epsps, gat*	Resistant to glyphosate
GM soybean	zhongliandou1307	zhonghuang6106	*g2-epsps, gat*	Resistant to glyphosate
GM soybean	zhongliandou2825	zhonghuang6106	*g2-epsps, gat*	Resistant to glyphosate
GM soybean	zhongliandou2109	zhonghuang6106	*g2-epsps, gat*	Resistant to glyphosate
GM soybean	zhongliandou2041	zhonghuang6106	*g2-epsps, gat*	Resistant to glyphosate

	Name of variety	Name of transformant	Transferred genes	GM traits
GM soybean	zhongliandou1309	zhonghuang6106	*g2-epsps, gat*	Resistant to glyphosate
GM soybean	zhongliandou1311	zhonghuang6106	*g2-epsps, gat*	Resistant to glyphosate
GM soybean	zhongliandou1510	zhonghuang6106	*g2-epsps, gat*	Resistant to glyphosate
GM soybean	zhongliandou1512	zhonghuang6106	*g2-epsps, gat*	Resistant to glyphosate
GM soybean	zhongliandou5046	zhonghuang6106	*g2-epsps, gat*	Resistant to glyphosate
GM soybean	zhongliandou6024	zhonghuang6106	*g2-epsps, gat*	Resistant to glyphosate
GM soybean	maiyu4003	DBN9004	*epsps, pat*	Resistant to glyphosate and glufosinate
